# Effects of *in ovo* Injection of *Astragalus* Polysaccharide on the Intestinal Development and Mucosal Immunity in Broiler Chickens

**DOI:** 10.3389/fvets.2021.738816

**Published:** 2021-08-30

**Authors:** Shu-bao Yang, Yan-jun Qin, Xin Ma, Wei-min Luan, Peng Sun, An-qi Ju, Ao-yi Duan, Ying-nan Zhang, Dong-hai Zhao

**Affiliations:** ^1^Basic Medical College, Jilin Medical University, Jilin, China; ^2^College of Animal Science and Technology, Jilin Agricultural University, Changchun, China; ^3^School of Public Health, Jilin Medical University, Jilin, China; ^4^College of Life Science, Changchun Sci-Tech University, Changchun, China

**Keywords:** *Astragalus* polysaccharide, *in ovo* injection, intestinal development, intestinal mucosal immunity, broilers

## Abstract

The purpose of this study was to examine the effects of *in ovo* injection of *Astragalus* polysaccharide (APS) on hatchability, body weight (BW), intestinal histomorphology, the number of IgA^+^ cells and sIgA content in intestine, and the expression of intestinal immune-related genes in broiler chickens. On day 18 of the incubation, a total of 960 live embryo eggs were weighed and randomly divided into 4 treatment groups: a control group and three APS groups. The eggs in the control group were injected with 0.5 mL physiological saline. The eggs in the APS groups were injected with 3 different amounts of APS in 0.5 mL physiological saline: 1 mg (APS_L_), 2 mg (APS_M_) and 4 mg (APS_H_). The solution was injected into the amnion of each egg. The results showed that *in ovo* injection of APS did not affect the hatchability but increased the body weight of the 14 d and 21 d chickens, with a significant increase observed in the APS_M_ group (*P* < 0.05). At most time points, the villus height (VH) was increased (*P* < 0.05) and the crypt depth (CD) was decreased (*P* < 0.05) in the small intestine of the broilers, with higher VH/CD ratios in the APS_L_ and APS_M_ groups compared with the control group. The number of IgA^+^ cells in the mucosa and the secretory immunoglobulin A (sIgA) levels in the intestinal washings were higher in the APS_M_ and APS_H_ groups than in the APS_L_ and control groups. The gene expression levels of interleukin (IL)-2, interleukin (IL)-4, interferon gamma (IFN-γ), and Toll-like receptor (TLR)-4 were significantly enhanced by APS stimulation at most time points (*P* < 0.05). These results indicated that *in ovo* injection of APS has the potential of promoting intestinal development and enhancing intestinal mucosal immunity of broiler chickens in the early stage after hatching.

## Introduction

In comparison to mammals, the growth and development of the avian embryo and hatchling are dependent on the nutrients in the fertile eggs. Due to avian embryos' rapid growth and high metabolism, they have a great demand for energy and nutrients during late embryonic development, resulting in an imbalance between the consumption and retention of nutrients within the eggs (1). In recent years, *in ovo* feeding during incubation is regarded as a way to correct this imbalance by providing additional nutrients to meet the developmental needs of embryos and post-hatch chickens during fasting (2, 3).

Newly hatched chickens have limited maternal antibodies and immature immune and digestion systems, which make them susceptible to infections. Early research showed that intestinal mucosa is a vital tissue that maintains nutrition and immunity in the body (4, 5). Therefore, strengthening the intestinal immune function to improve resistance against external pathogens plays a key role in the survival and development of young chickens (6). Newly hatched chicks are restricted in their access to feed for ~48–72 h in commercial poultry rearing. As studies indicated the fasting may lead to retardation in development of the gastrointestinal tract and even the entire body (7, 8). Studies have shown that *in ovo* injection of various biologics into the amnion, such as prebiotics and synbiotics, may rapidly promote small intestinal growth and development by establishing a healthy and balanced gastrointestinal tract (GIT) microbiome in the early stage of its formation (9, 10).

Astragalus is a kind of Chinese medicinal herb that has been used in oriental medicine for thousands of years. *Astragalus* polysaccharide (APS) is a type of water-soluble heteropolysaccharide with bioactive effects, which is extracted from the stems or dried roots of Astragalus. The components are complex and diverse, and polymeric carbohydrates are mainly linked by a-type glycosidic bonds between the monosaccharides (11). *Astragalus* polysaccharide (APS) is the main ingredient extracted from Astragalus, which has multiple biological activities, including immunomodulatory, anti-viral, anti-tumor, and anti-oxidant properties (12). Previous studies indicated that APS possesses the effect of promoting intestinal development and modulating intestinal mucosal immunity of chicks when taken orally or as a feed additive (13, 14). The modulatory effect of APS on intestinal mucosal immunity in broilers is considerably dependent on the interaction with various immunoglobulins (e.g., IgA, IgG) and cytokines (e.g., IL-2, IL-4, and IL-6) when foreign antigen is present in the body of the broilers (15–17). However, little is known about the effect of *in ovo* injection of APS on the intestinal development and intestinal mucosal immunity in broiler chicks.

Therefore, the aim of the present study was to evaluate the effect of *in ovo* injection with 3 different amounts of APS on the intestinal development and mucosal immunity in broilers by determinations of hatchability, body weight, intestinal histomorphology, the distribution and number of IgA^+^ cells, sIgA levels, and the expression of intestinal immune-related genes. The information obtained in this study can therefore be useful for understanding effects of *in ovo* injection of APS on growth and immunity adjustment in poultry industry.

## Materials and Methods

### Experimental Design and Egg Incubation

This study was conducted following the Jilin Agriculture University Institutional Animal Care and Use Committee (JLAU08201409), and the experimental procedures were performed in compliance with the National Institutes of Health Guide for the Care and Use of Laboratory Animals (NIH Publications No. 8,023).

Fertilized Arbor Acres (AA+) broiler eggs were obtained from a broiler breeder farm (Runcheng Broiler Breeding Factory, Changchun, China). All eggs with a similar weight (60.27 ± 0.15 g) were incubated under standard conditions in a Hongtai incubator (Hongtai Incubation Equipment Factory, China). On days 12 and 18 of incubation, the eggs were candled and the unfertilized eggs and dead embryos were discarded. A total of 960 live embryo eggs were weighed and randomly allocated to 4 treatment groups (a control group and three APS groups), consisting of 3 replicates of 80 eggs each on day 18 of incubation. APS (net content 91.9%) was purchased from Sihai Plant Extracts Co., Ltd. (Nantong, China). The control group was injected with 0.5 mL physiological saline, while the three experimental groups (APS_L_, APS_M_, and APS_H_ group) were injected with 0.5 mL of 3 different concentrations of APS solution (1, 2, and 4 mg APS in 0.5 mL physiological saline), respectively. The saline and APS solutions were injected into the amnion of each egg vertically from the top of the larger end of the eggshell to a depth of 2.49 cm using a 22-gauge needle. A new needle was used for each injection. After *in ovo* injection, the holes were sealed with sterile paraffin and the eggs were returned to the incubator. Each tray of eggs of each treatment group remained outside the incubator for ~15 min during the injection period.

### Animal Treatment and Sample Collection/Data Collection

On the day of hatching, the hatchability of the injected live embryonated eggs was calculated, and all male hatched chicks from one treatment were pooled and weighted. A total of 75 male chicks from each of the four experimental treatments with a similar weight close to the average body weight (BW) of their pooled group were selected and randomly assigned into 3 replicates of 25 chicks each. A total of 12 cages were provided for the four treatments, with each replicate allocated to a cage. Chicks were reared up to 21 d of age and were offered water and a standard broiler diet ad libitum. On days 1, 7, 14, and 21 post-hatch, five birds per replicate were weighed and euthanized by cervical dislocation, and the duodenum, jejunum, and ileum were separated. A hematoxylin and eosin (H&E) staining method was used to observe changes in the intestinal morphological structure, and the distribution and quantity of IgA^+^ cells in the intestinal mucosa were studied by immunohistochemical staining. The changes in sIgA content in jejunal washing were detected by an enzyme-linked immunosorbent assay (ELISA). Real-time PCR was used to detect the expression of IL-2, IL-6, IFN-γ, and TLR-4 mRNA in the ileum.

### Histomorphological Measurements

Intestinal segments (duodenum, jejunum, and ileum) were fixed in 10% formalin, embedded in paraffin, then cut into 6-μm thick sections (Typ RM 2235, Leica, Germany), and stained using a hematoxylin and eosin (H&E) method. Five villi and crypts for 5 sections per sample in each chicken from each group were measured and analyzed using Image-Pro Plus 6.0 software (Media Cybernetics, USA). The villus height (VH) was measured from the top of the villus to the crypt mouth, the depth of the invagination between adjacent crypt mouths was measured and defined as crypt depth (CD), and the ratio of VH/CD was calculated.

### Analysis of Intestinal IgA^+^ Cells

The segments of duodenum and jejunum embedded with paraffin wax were cut into 6-μm thin sections with sledge microtome (Typ RM 2235, Leica, Germany), the paraffin was removed prior to staining with xylol, and then the sections were incubated with citrate-buffered solution (pH 6.0, 0.01 M) at 95°C for 20 min. The distribution and numbers of IgA^+^ cells in the duodenum and jejunum were identified with an immunohistochemistry staining method described previously (14). The reactions were made visible with metal-enhanced diaminobenzidine (DAB). After staining, the sections were slightly counterstained with hematoxylin. The staining sections were observed under 100× amplification (Olympus CX41 microscope and the Pixera pro600ES image acquisition unit, Pixera, USA) and measured using Image-pro plus 6.0 software (Media Cybernetics, USA). The IgA^+^ cells of five views from each slice from five sections per group were counted, and the average numbers of IgA^+^ cells per view (over areas of 0.01 mm^2^) for each group were calculated.

### Detection of SIgA Content in Jejunum

As described by Shan et al. (14), 5-cm sections of the jejunum were removed, infused with 0.5 mL PBS (pH 7.4, containing 0.1% BSA and aprotinin) and washed three times. The washings were collected and centrifuged at 10,950 × g at 4°C for 10 min. The supernatants were collected to determine the sIgA levels in the jejunum mucosa using an ELISA according to the instructions of the Chicken sIgA Kit-BPE60021 (Lengton Bioscience Co., Ltd., Shanghai, China). Optical density was measured using a microplate reader (Sunrise-Basic, Switzerland) at 450 nm.

### Real-Time Quantitative RT-PCR (qRT-PCR)

The ileum samples were removed, and 20–30 mg aliquots were weighed. The total RNA of the ileum was extracted according to the instruction of the RNA simple Total RNA kit-DP431 (Tiangen Biotechnology Co., Ltd., Beijing, China) and reverse transcribed into cDNA using the PrimeScript TM Reagent Kit with gDNA Eraser (DRR047A) (Tiangen Biotechnology Co., Ltd., Beijing, China). The amplification and detection were conducted using the SYBR® Premix Ex TaqTM II Real-time RT-PCR (qRT-PCR) kit (Bao Bioengineering Co., Ltd., Dalian, China) in an Applied Biosystems 7500 FAST Real-Time PCR System. During the PCR, the samples were subjected to an initial denaturation phase at 95°C for 20 s, followed by 40 cycles of denaturation at 95°C for 3 s, and annealing and extension at 60°C for 30 s. The gene expression of IL-2, IL-6, IFN-γ, and TLR-4 was analyzed using β-actin as an endogenous control. The forward and reverse primer sequences are listed in Table 1. We obtained the relative gene expression level using the 2^−ΔΔCT^ method. All PCR operations were performed in triplicate.

**Table 1:** Specific primers for β-actin, IL-2, IL-6, IFN-γ, and TLR-4.

**Gene**	**Primer**	**Sequence**	**Amplicon size (bp)**
β-actin	Forward	TGATATTGCTGCGCTCGTTG	143
	Reverse	CTTTCTGGCCCATACCAACC	
IL-2	Forward	CAAGAGTCTTACGGGTCTAAATCAC	100
	Reverse	GTTGGTCAGTTCATGGAGAAAATC	
IL-6	Forward	CAAGGTGACGGAGGAGGAC	254
	Reverse	TGGCGAGGAGGGATTTCT	
IFN-γ	Forward	GACAAGTCAAAGCCGCACA	127
	Reverse	TCAAGTCGTTCATCGGGAGC	
TLR-4	Forward	CCACACACCTGCCTACATGAA	190
	Reverse	GGATGGCAAGAGGACATATCAAA	

### Statistical Analysis

Data were analyzed using SPSS 22.0 (SPSS Inc., Cary, NC, USA). One-way analysis of variance (ANOVA) with a Duncan *post-hoc* test was used for multiple comparisons among different groups. The overall data are expressed as the mean ± SEM, and significant differences were considered at *P* < 0.05.

## Results

### Hatchability and Post-hatch BW

There were no significant differences in hatchability among the four groups (*P* > 0.05, Table 2). BW were not affected by *in ovo* treatment on days 1 and 7 post-hatch, and no significant differences were observed among the four groups (*P* > 0.05). However, on d 14 and d 21, the BW in the APS_M_ group was significantly higher than those in the other three groups (*P* < 0.05, Table 2).

**Table 2:** Effect of *in ovo* injection of APS on hatchability and BW.

**Item**	**Treatment groups**				***P*-value**
	**Control**	**APS_**L**_**	**APS_**M**_**	**APS_**H**_**	
Hatchability (%)	90.0 ± 2.55	88.9 ± 1.89	91.1 ± 2.97	87.8 ± 3.11	
**BW (g)**					
d 1	47.21 ± 0.87	46.32 ± 2.68	48.11 ± 1.36	47.32 ± 1.61	0.092
d 7	151.35 ± 2.42	156.72 ± 5.94	150.22 ± 3.67	160.25 ± 3.53	0.072
d 14	415.23 ± 7.85^b^	420.11 ± 11.46^b^	435.14 ± 10.80^a^	411.28 ± 9.45^b^	0.023
d 21	691.44 ± 12.61^b^	707.26 ± 18.02^b^	738.33 ± 15.34^a^	711.28 ± 19.97^b^	0.031

*Control group, 0.5 mL physiological saline; APS_L_ group, low dose of APS group (2 mg/ml); APS_M_, middle dose of APS group (4 mg/ml); APS_H_, high dose of APS group (8 mg/ml). The results are reported as the means ± SEM*.

a,b *Means with different superscripts within the same column differ significantly (P < 0.05)*.

### Intestinal Histomorphological Analyses

**Figure 1:**
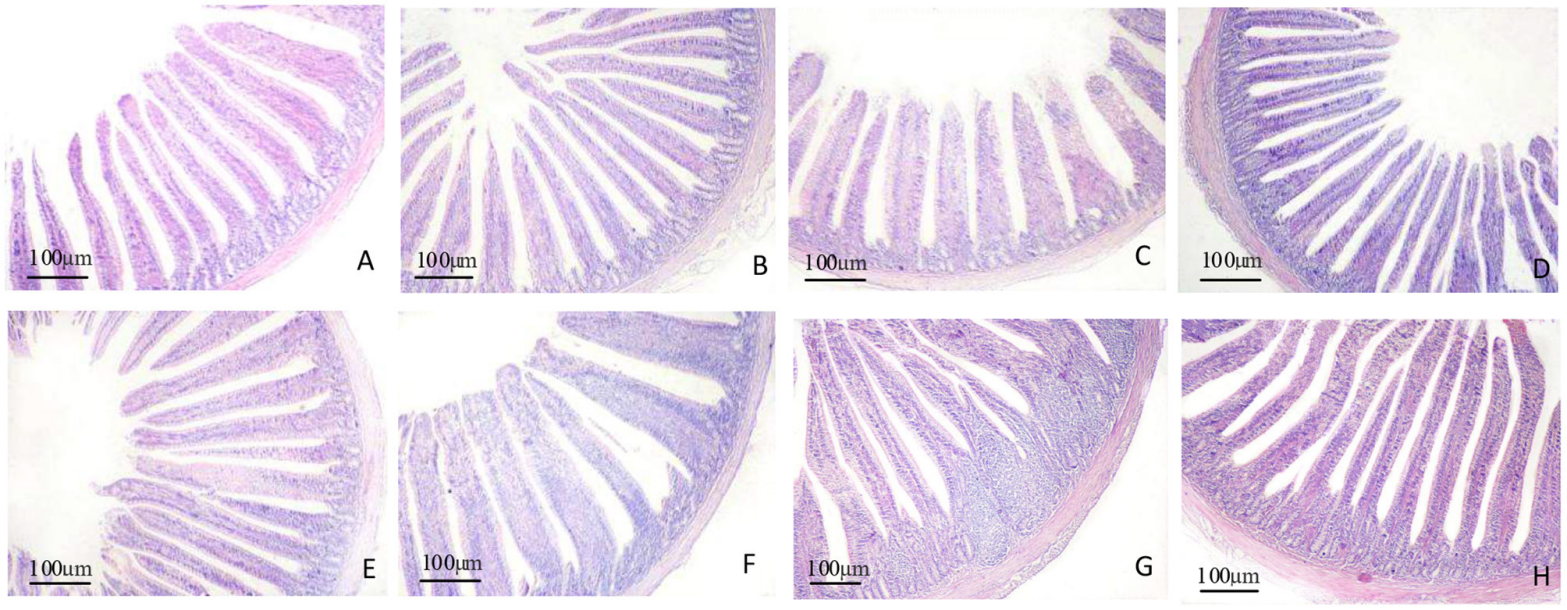
Representative light microscopic histological views of duodenal morphological structure in different groups (HE staining). Control group, 0.5 mL physiological saline; APS_L_ group, low dose of APS group (2 mg/ml); APS_M_, middle dose of APS group (4 mg/ml); APS_H_, high dose of APS group (8 mg/ml). **(A,B)** The control and APS_M_ group on d 7; **(C–E)** the control, APS_M_ and APS_H_ group on d 14; **(F–H)** the control, APS_M_ and APS_H_ group on d 21.

**Figure 2:**
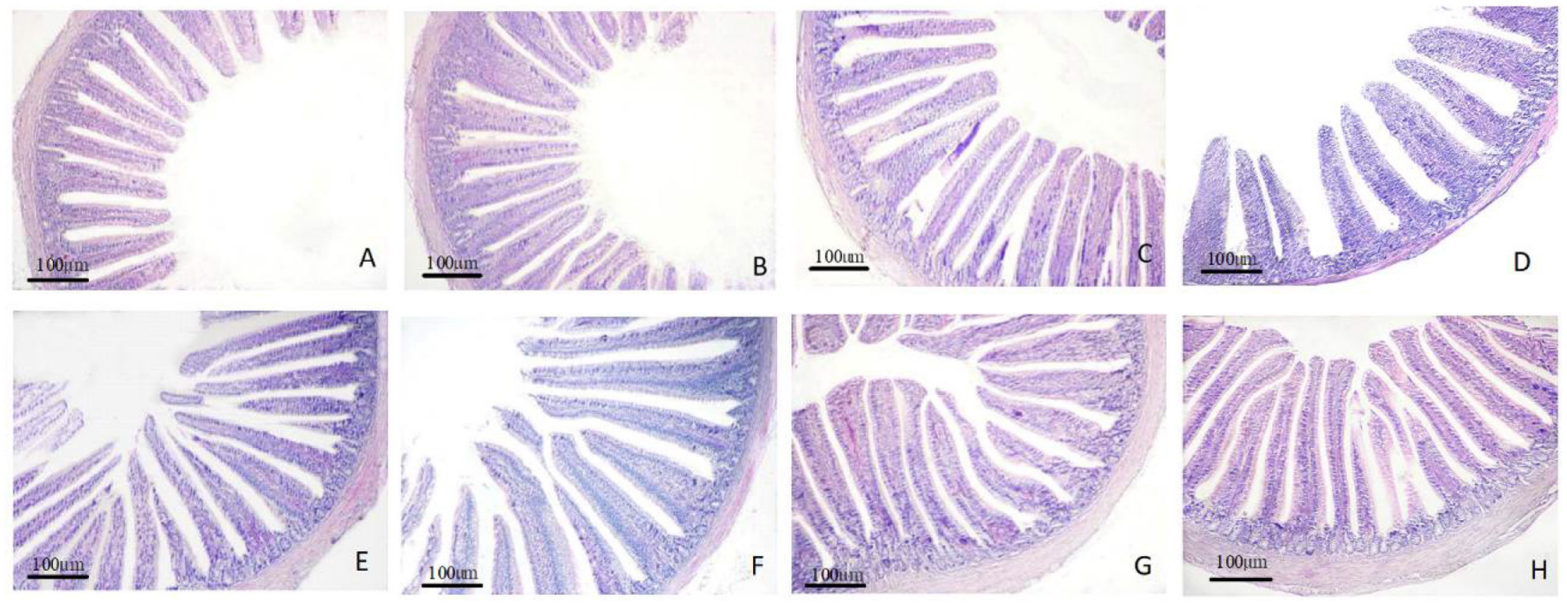
Representative light microscopic histological views of jejunum morphological structure in different groups (HE staining). Control group, 0.5 mL physiological saline; APS_L_ group, low dose of APS group (2 mg/ml); APS_M_, middle dose of APS group (4 mg/ml); APS_H_, high dose of APS group (8 mg/ml). **(A–C)** The control, APS_L_ and APS_M_ group on d 7; **(D,E)** the control and APS_L_ group on d 14; **(F–H)** the control, APS_L_ and APS_M_ group on d 21.

**Figure 3:**
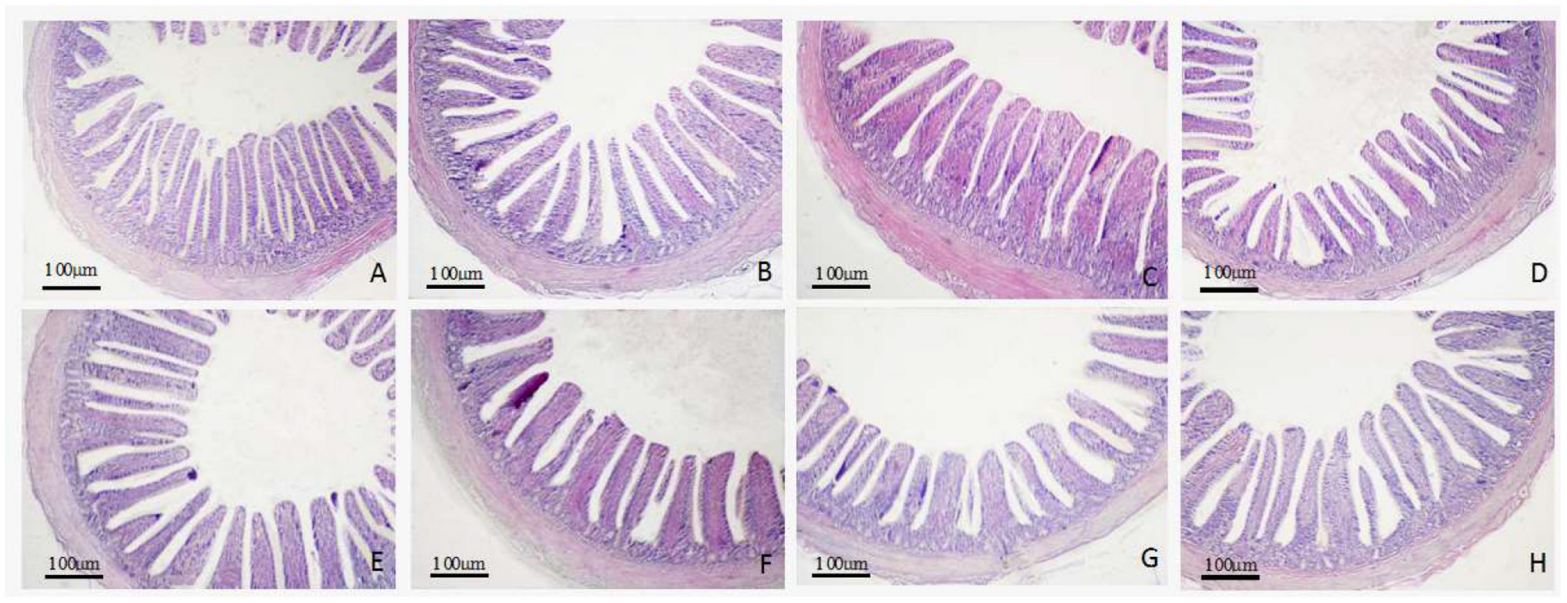
Representative light microscopic histological views of ileum morphological structure in different groups (HE staining). Control group, 0.5 mL physiological saline; APS_L_ group, low dose of APS group (2 mg/ml); APS_M_, middle dose of APS group (4 mg/ml); APS_H_, high dose of APS group (8 mg/ml). **(A–C)** The control, APS_L_ and APS_H_ group on d 7; **(D–F)** the control, APS_L_ and APS_M_ group on d 14; **(G,H)** the control, APS_L_ group on d 21.

The morphology changes in the duodenum, jejunum, and ileum are shown in Figures 1–**3**. Compared to the control group, the duodenal villi were in a closer and more orderly array on d 7 and d 14. On d 21, the duodenal glands were better developed in the three APS groups (Figure 1). With increasing age, the jejunal villi in the three APS groups grew longer and their arrangement was closer than those in the control group (Figure 2). The villi of the ileum from the APS groups were longer and wider than those in the control group (Figure 3).

The VH, CD, and the ratio of VH/CD of duodenum, jejunum, and ileum are shown in **Tables 3–5**. In the duodenum, there were no significant differences in the VH among the 4 treatment groups on d 1 (*P* > 0.05, Table 3). The VH of duodenum significantly increased in the APS_M_ and APS_H_ groups on d 7 and d 21 when compared to the control group (*P* < 0.05, Table 3). However, there was no significant increase in VH, CD, and VH/CD in the APS_L_ group on d 14 and d 21 in comparison with the control group (*P* > 0.05, Table 3). On d 21, the VH and CD of duodenum were highest in the APS_M_ group among three APS groups and were significantly higher than those in the control group (*P* < 0.05, Table 3).

**Table 3:** Effect of *in ovo* injection of APS on duodenum morphology at different ages.

**Item**	Treatment groups^*^				***P*-value**
	**Control**	**APS_**L**_**	**APS_**M**_**	**APS_**H**_**	
**Villus height (VH**, **μm)**					
d 1	543.76 ± 28.74	537.29 ± 20.16	542.42 ± 47.40	543.31 ± 39.35	0.108
d 7	676.16 ± 30.95^c^	753.16 ± 13.97^b^	882.03 ± 36.77^a^	738.66 ± 27.54^b^	0.011
d 14	786.23 ± 13.87^b^	775.16 ± 22.45^b^	862.36 ± 14.49^a^	778.53 ± 17.11^b^	0.013
d 21	866.43 ± 48.76^c^	901.30 ± 46.8^c^	1063.76 ± 32.88^a^	953.40 ± 17.13^b^	0.025
**Crypt depth (CD**, **μm)**					
d 1	100.33 ± 10.99^a^	36.74 ± 3.03^c^	55.16 ± 6.79^b^	47.46 ± 3.75^bc^	0.004
d 7	122.53 ± 21.71	101.00 ± 2.80	102.4333 ± 8.35	105.73 ± 7.28	0.132
d 14	115.2 ± 2.92^a^	95.06 ± 17.65^b^	114.3 ± 9.77^a^	114.23 ± 4.30^a^	0.018
d 21	103.06 ± 8.48^b^	100.80 ± 8.60^b^	144.5 ± 13.71^a^	140.96 ± 15.16^a^	0.027
**VH/CD**					
d 1	5.43 ± 0.33^c^	14.23 ± 0.93^a^	9.82 ± 1.09^b^	11.52 ± 1.34^ab^	0.004
d 7	6.62 ± 0.07^b^	7.46 ± 0.33^ab^	8.67 ± 1.04^a^	7.01 ± 0.73^b^	0.002
d 14	6.83 ± 0.29	8.34 ± 1.55	7.58 ± 0.70	6.82 ± 0.37	0.112
d 21	6.82 ± 0.75^b^	7.40 ± 0.80^b^	8.46 ± 0.65^a^	8.53 ± 0.89^a^	0.015

*Control group, 0.5 mL physiological saline; APS_L_ group, low dose of APS group (2 mg/ml); APS_M_, middle dose of APS group (4 mg/ml); APS_H_, high dose of APS group (8 mg/ml). The results are reported as the means ± SEM*.

a−c *Means with different superscripts within the same column differ significantly (P < 0.05). VH/CD, the ratio of VH to CD*.

* *A total of six replicates were used per treatment*.

In the jejunum, no significant differences of the VH, CD, and the ratio of VH/CD among the 4 treatment groups on d 1 were observed (*P* > 0.05, Table 4). At most time points, the VHs of the APS groups were significantly higher than those in the control groups (*P* < 0.05, Table 4), but there were no significant differences in the CDs except for the APS_L_ group on d 21 (*P* > 0.05, Table 4). The VH/CD ratios of the APS_H_ group on d 7 and the APS_M_ group on d 21 were significantly higher than those in the control group (*P* < 0.05, Table 4).

**Table 4:** Effect of in *ovo* injection of APS on jejunum morphology at different ages.

**Item**	Treatment groups^*^				***P*-value**
	**Control**	**APS_**L**_**	**APS_**M**_**	**APS_**H**_**	
**Villus height (VH**, **μm)**					
d 1	425.36 ± 47.12	415.70 ± 6.08	417.40 ± 26.70	413.36 ± 4.93	0.086
d 7	524.76 ± 20.54^c^	668.80 ± 11.70^a^	575.76 ± 9.11^b^	567.60 ± 36.07^b^	0.007
d 14	732.70 ± 65.40^b^	731.90 ± 18.06^b^	1079.10 ± 15.92^a^	740.93 ± 15.89^b^	0.011
d 21	1007.33 ± 77.01^c^	1140.96 ± 26.78^b^	1416.20 ± 72.08^a^	942.23 ± 13.99^c^	0.018
**Crypt depth (CD**, **μm)**					
d 1	48.93 ± 2.72^a^	49.00 ± 1.60^a^	47.56 ± 2.12^a^	38.60 ± 6.50^b^	0.027
d 7	80.50 ± 8.22	88.86 ± 4.30	89.10 ± 14.00	83.60 ± 11.83	0.098
d 14	126.73 ± 22.56^ab^	106.73 ± 5.54^b^	145.43 ± 8.34^a^	105.96 ± 5.73^b^	0.009
d 21	163.00 ± 7.66^b^	188.30 ± 21.27^a^	171.46 ± 7.24^b^	162.96 ± 6.18^b^	0.004
**VH/CD**					
d 1	4.27 ± 0.61	4.00 ± 0.29	4.41 ± 0.46	5.03 ± 0.81	0.135
d 7	5.41 ± 0.54^b^	5.15 ± 0.57^b^	5.55 ± 0.85^b^	6.76 ± 0.16^a^	0.035
d 14	5.92 ± 0.96^b^	6.86 ± 0.18^ab^	7.44 ± 0.46^a^	6.34 ± 0.44^ab^	0.027
d 21	6.17 ± 0.17^b^	6.14 ± 1.13^b^	8.27 ± 0.62^a^	5.79 ± 0.31^b^	0.014

*Control group, 0.5 mL physiological saline; APS_L_ group, low dose of APS group (2 mg/ml); APS_M_, middle dose of APS group (4 mg/ml); APS_H_, high dose of APS group (8 mg/ml). The results are reported as the means ± SEM*.

a−c *Means with different superscripts within the same column differ significantly (P < 0.05). VH/CD, the ratio of VH to CD*.

* *A total of six replicates were used per treatment*.

In the ileum, the VHs in the APS_M_ and APS_H_ group on d 1 and d 7 were significantly higher in comparison with the control group and APS_L_ group (*P* < 0.05, Table 5). The CDs of the ileum significantly increased in the three APS treatment groups when compared to the control group on d 21 (*P* < 0.05, Table 5). On d 14 and d 21, the ratios of VH/CD in the APS_L_ group were significantly higher than those in the control group (*P* < 0.05, Table 5).

**Table 5:** Effect of in *ovo* injection of APS on ileum morphology at different ages.

**Item**	Treatment groups^*^				***P*-value**
	**Control**	**APS_**L**_**	**APS_**M**_**	**APS_**H**_**	
**Villus height (VH**, **μm)**					
d 1	395.33 ± 8.00^b^	417.70 ± 25.46^b^	460.10 ± 13.48^a^	461.26 ± 10.31^a^	0.007
d 7	488.86 ± 8.52^c^	543.70 ± 4.27^b^	565.76 ± 12.79^a^	579.66 ± 6.99^a^	0.021
d 14	429.53 ± 24.15^b^	492.30 ± 13.78^a^	449.36 ± 40.19^ab^	464.36 ± 8.33^ab^	0.025
d 21	383.26 ± 10.15^b^	512.60 ± 16.20^a^	411.50 ± 14.22^b^	398.10 ± 9.73^b^	0.017
**Crypt depth (CD**, **μm)**					
d 1	94.10 ± 9.58	104.40 ± 3.63	105.26 ± 13.98	93.20 ± 14.54	0.109
d 7	90.76 ± 7.43	106.33 ± 11.57	101.86 ± 3.54	85.73 ± 2.70	0.112
d 14	92.46 ± 7.59^a^	93.56 ± 4.06^a^	84.00 ± 13.61^b^	89.83 ± 7.76^a^	0.019
d 21	66.63 ± 1.66^c^	75.60 ± 4.99^b^	95.06 ± 2.70^a^	83.26 ± 5.46^b^	0.024
**VH/CD**					
d 1	4.27 ± 0.66	4.00 ± 0.28	4.41 ± 0.46	5.03 ± 0.81	0.103
d 7	5.41 ± 0.54^b^	5.15 ± 0.57^b^	5.55 ± 0.08^b^	6.76 ± 0.16^a^	0.009
d 14	4.65 ± 0.26^bc^	5.26 ± 0.20^a^	5.39 ± 0.45^a^	4.32 ± 0.36^c^	0.001
d 21	5.75 ± 0.23^b^	6.81 ± 0.67^a^	5.33 ± 0.15^b^	5.79 ± 0.21^b^	0.005

*Control group, 0.5 mL physiological saline; APS_L_ group, low dose of APS group (2 mg/ml); APS_M_, middle dose of APS group (4 mg/ml); APS_H_, high dose of APS group (8 mg/ml). The results are reported as the means ± SEM*.

a−c *Means with different superscripts within the same column differ significantly (P < 0.05). VH/CD: the ratio of VH to CD*.

* *A total of six replicates were used per treatment*.

### IgA^+^ Cells in the Duodenal and Jejunal Mucosa

**Figure 4:**
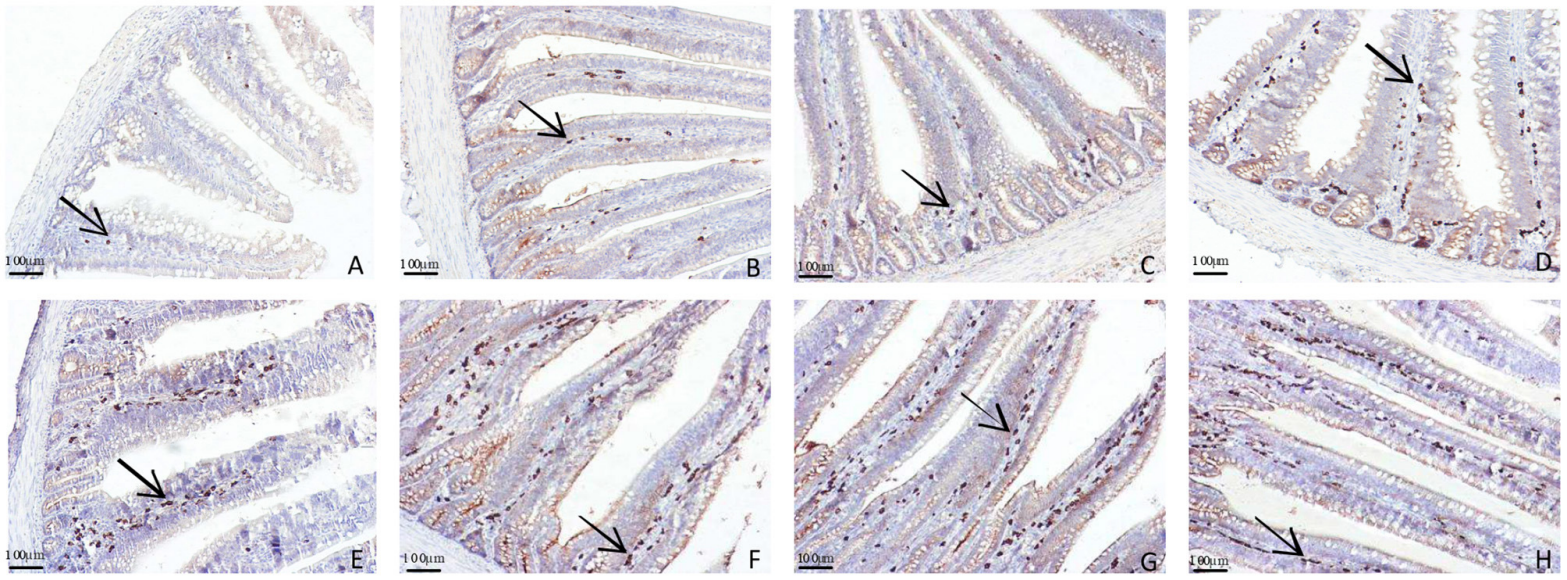
Representative light microscopic histological views of IgA^+^ cells in the duodenal mucosa in different groups (arrow: IgA^+^ positive cells). Control group, 0.5 mL physiological saline; APS_L_ group, low dose of APS group (2 mg/ml); APS_M_, middle dose of APS group (4 mg/ml); APS_H_, high dose of APS group (8 mg/ml). **(A)** The control group on d 1; **(B,C)** the control and APS_H_ group on d 7; **(D,E)** the control and APS_H_ group on d 14; **(F–H)** the control, APS_M_ and APS_L_ group on d 21.

**Figure 5:**
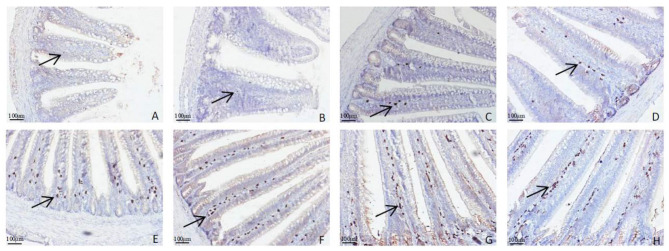
Representative light microscopic histological view of IgA+ cells in the jejunal mucosa in different groups (arrow: IgA^+^ positive cells). Control group, 0.5 mL physiological saline; APS_L_ group, low dose of APS group (2 mg/ml); APS_M_, middle dose of APS group (4 mg/ml); APS_H_, high dose of APS group (8 mg/ml). **(A,B)** The control and APS_L_ group on d 1; **(C,D)** the control and APS_M_ group on d 7; **(E,F)** the control and APS_M_ group on d 14; **(G,H)** the control and APS_M_ group on d 21.

**Figure 6:**
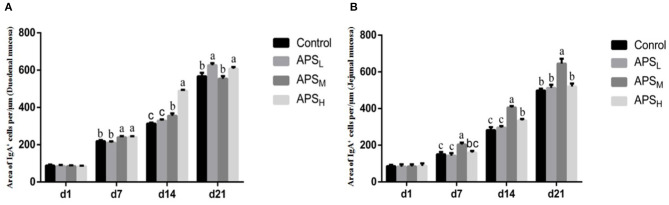
Effects of APS on IgA^+^ cell development in the duodenal villus **(A)** and the jejunal villus **(B)**. The results are reported as the means ± SEM. Control group, 0.5 mL physiological saline; APSL group, low dose of APS group (2 mg/ml); APSM, middle dose of APS group (4 mg/ml); APSH, high dose of APS group (8 mg/ml). Different letters within a column indicate significant difference among 4 treatment groups (*P* < 0.05). d, days post-hatch.

The distributions of IgA^+^ cells in the duodenal and jejunal mucosa are shown in Figures 4, 5. The numbers of IgA^+^ cells in the duodenal and jejunal mucosa are shown in Figures 6A,B. On d 1, IgA^+^ cells were scattered in the lamina propria of the intestinal villi (Figure 4A), and the numbers of IgA^+^ cells in the duodenum in all groups showed no significant differences (*P* > 0.05; Figure 6A). Moreover, no obvious differences in the IgA^+^ cell distribution were observed among the 4 treatment groups in the duodenal mucosa. On d 7 and d 14, more IgA^+^ cells were distributed in the lamina propria of the intestinal villi and around the intestinal glands in the APS_H_ group compared to the control group (Figures 4B–E). On d 21, more IgA^+^ cells were observed in the lower and middle parts of the lamina propria and at the bottom of the duodenal mucosa in the APS_M_ and APS_H_ groups (Figures 4F–H). On d 7–21, the numbers of IgA^+^ cells in the APS_M_ and APS_H_ groups in the duodenum were significantly higher than those in the control group (*P* < 0.05), but a significant difference was not observed between the APS_L_ group and the control group on d 7 and d 14 post-hatch (*P* > 0.05, Figure 6A).

The distribution of IgA^+^ cells in the jejunal mucosa was similar to the duodenal mucosa at each time point, but there were less IgA^+^ cells in the bottom of the jejunal mucosa and around the intestinal gland in the jejunal mucosa than those in the duodenal mucosa (Figures 4D–H, 5A–H). The numbers of IgA^+^ cells in the APS_M_ group were significantly higher than those in the APS_L_, APS_H_, and control group on d 7 in the jejunum (*P* < 0.05, Figure 6B). On d 14 and d 21, the IgA^+^ cell numbers in the APS_M_ group were significantly higher than those in the control group and the APS_L_ group (*P* < 0.05, Figure 6B).

### Changes in SIgA Levels

**Figure 7:**
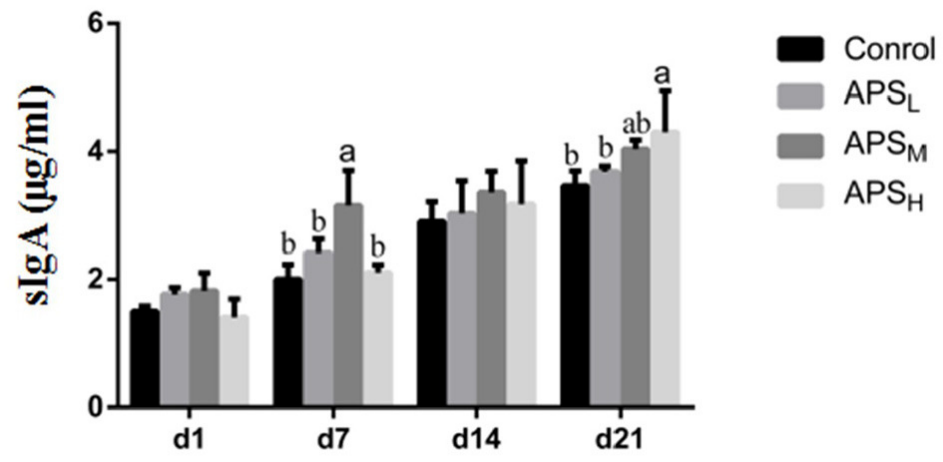
Effect of *in ovo* injection of APS on sIgA levels in jejunum contents. The results are reported as the means ± SEM. Control group, 0.5 mL physiological saline; APSL group, low dose of APS group (2 mg/ml); APSM, middle dose of APS group (4 mg/ml); APSH, high dose of APS group (8 mg/ml). Different letters within a column indicate significant difference among 4 treatment groups (*P* < 0.05).

The effects of APS on sIgA levels in jejunum washings are shown in Figure 7. There were no significant differences in sIgA levels on d 1 and d 14 among the four groups (*P* > 0.05). Compared to the control group, the sIgA levels in the APS_M_ group significantly increased on d 7 and d 21 (*P* < 0.05). On d 21, the level of sIgA in the APS_H_ group was the highest among the four groups and was significantly higher than that in the control group and APS_L_ group (*P* < 0.05).

### Expression Levels of Intestinal Immune-Related Genes

**Figure 8:**
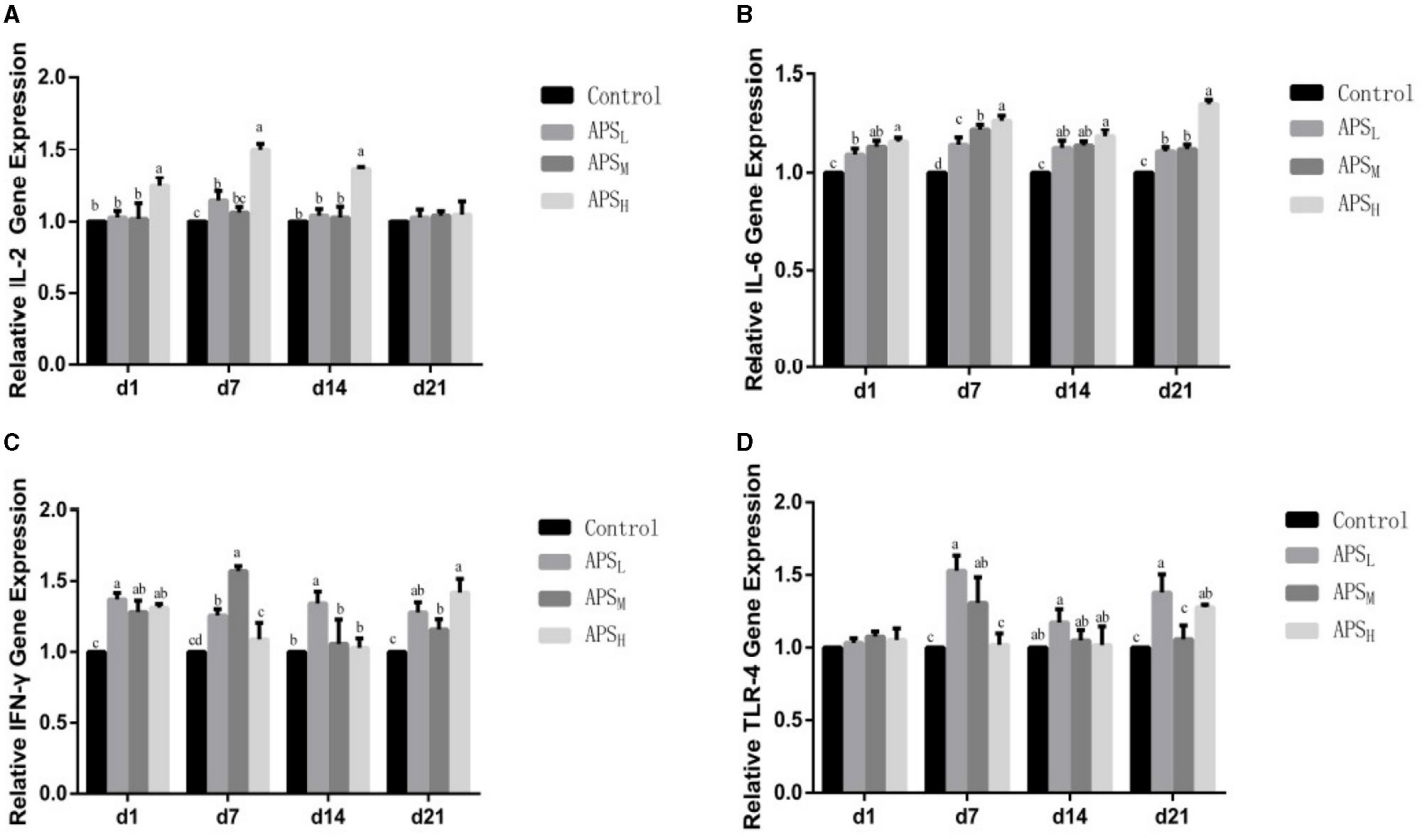
Effect of *in ovo* injection of APS on the relative expression of IL-2, IL-6, IFN-γ, and TLR-4 mRNA in the ileum of different ages. **(A)** IL-2; **(B)** IL-6; **(C)** IFN-γ; **(D)** TLR-4. The results are reported as the means ± SEM. Control group, 0.5 mL physiological saline; APS_L_ group, low dose of APS group (2 mg/ml); APS_M_, middle dose of APS group (4 mg/ml); APS_H_, high dose of APS group (8 mg/ml). Different letters within a column indicate significant difference among 4 treatment groups (*P* < 0.05).

The relative gene expression levels of IL-2, IL-6, IFN-γ, and TLR-4 in the ileum were examined, and the results are shown in Figure 8. In comparison with the control group, the expression levels of IL-2 were significantly upregulated in the APS_H_ group on d 1, 7, and 14 (*P* < 0.05, Figure 8A). The IFN-γ expression levels were significantly upregulated in the APS_M_ group on d 7 and d 14 and significantly upregulated in the APS_L_ group at all time points (*P* < 0.05, Figure 8C). In addition, there were no significant differences in the expression of IL-2 on d 1 and d 7 among the three APS groups (*P* > 0.05, Figures 8A,C). The expression levels of IL-6 were significantly higher in the APS_M_ group and APS_H_ group (especially in the APS_H_ group) than those in the control group at each time point (*P* < 0.05, Figure 8B). Compared to the control group, the relative expression of TLR-4 in the APS_L_ group was higher than those in the control group on d 7 and d 21 (*P* < 0.05, Figure 8D).

## Discussion

Since the administration by *in ovo* delivery was first reported several decades ago, various biologics, such as carbohydrates, probiotics, and synbiotics, have been injected into embryos during the incubation period for promoting the health and productivity of poultry (1, 18). This study aimed to explore the effects of intra-amniotic injection of APS on growth performance, intestinal development and intestinal mucosal immunity.

In this experiment, *in ovo* supplementation of APS increased the BW without a negative effect of hatchability. These findings are consistent with the results reported in previous studies that both the injection of growth hormone into the albumen and the injection of lactic acid bacteria into the amnion did not affect hatchability (19, 20). Similarly, the injection of probiotics and drugs into the amnion did not affect hatchability or hatching weight (21, 22). However, other researchers have found that some carbohydrates and amino acids have been found to lead to a significantly reduced hatchability (23, 24). Retes et al. (25) believes that the hatchability may depend on egg size, inoculation, the volume of inoculated solution, and osmolarity. The findings of this study are suggesting that the APS can be safely administered *in ovo* without negatively affecting hatchability.

The small intestine is highly specialized in the hydrolysis and absorption of nutrients and constitutes the barrier between the host's external and internal environment (26, 27). The morphological indexes in the small intestine, such as the VH, CD, and VH/CD ratio, are important indicators of its health and functional status (28). The results in this experiment showed that the intra-amniotic injection of APS improved the morphological development of the small intestine on d 7–d 21, as indicated by an increase in VH and the ratio of VH/CD. The promoting effects of APS in the small intestine varied with the APS concentration, time point evaluated after hatch and the intestinal segment. However, it was not feasible to compare the data obtained from this experiment with the data from the literature, as most of those studies have been focused on the impact of APS by dietary inclusion that was not administered *in ovo* (29). In other studies, the VH and CD of the intestine were increased due to the intra-amniotic injection of nutrients (30, 31). Increased VH and CD enhance absorption and digestive capabilities (32). It was speculated that the early feeding of APS in the present study could increase the yolk-reserve utilization to enhance the small intestine growth and development in the late stage of hatching. Meanwhile, these surplus nutrients would continue to be utilized by the hatchling chicks during the fasting period.

Intestinal mucosal immunity is the first barrier against pathogen invasion in chickens, with more than 70% of immune cells (T cells, B cells, macrophages) located in the intestinal mucosa (33). The intestinal lamina propria contains abundant B lymphocytes, especially IgA^+^ cells. These IgA^+^ cells form an important mucosal protective layer on the surface of the intestinal mucosal and play an important role in protecting the intestinal tract from pathogenic infection. The sIgA produced by activated B cells is the most important factor in the mucosal adaptive immune system, forming a protective layer on the intestinal mucosal surface, and requires cytokines with immunomodulatory activity to guard against the incursion of harmful pathogens (34). The results of this study showed that APS delivered *in ovo* did not influence the number of IgA^+^ cells and the sIgA content on d 1 post-hatch. However, APS could increase the number of IgA^+^ cells in the intestinal mucosa and the sIgA content in the intestinal washings from d 7 to d 21. The ideal time period for injection was late-term avian embryo with delivery to the amniotic fluid. The embryo consumes the amniotic fluid and its contents are exposed to the intestines and the enteric cells that comprise them. Therefore, APS administered to this region will be consumed along with the amniotic fluid and presented to enteric tissues, and further enhanced intestinal mucosal immunity.

This study demonstrates that APS administration could increase the relative mRNA expression of IL-2, IL-6, IFN-γ, and TLR-4. As an important member of the Toll-like receptor family, TLR-4 can recognize microbial-associated molecular patterns of expression of infectious agents and plays an important role in the detection of pathogens. Increased TLR-4 is associated with an enhanced gastroepithelial barrier, which provides defense against pathogen invasion and infection (35). IL-6 is a key ingredient in cytokine network, not only participates in the inflammatory response, but also promotes the production of immune globulin. IL-2 and IFN-γ are important cytokines that play a fundamental role in stimulating the proliferation of B lymphocytes and T lymphocytes by inhibiting the production of pro-inflammatory modulators (36). The results in this experiment showed that the three doses of APS could have different effects on the gene expression of IL-2, IL-6, IFN-γ, and TLR-4. *In ovo* injection with APS_H_ had the most obvious positive effect on the gene expression of IL-2 and IL-6, and the gene expression of TLR-4 was most significantly increased at d 14 and d 21 in the APS_L_ group. However, the three doses of APS had different effects on the increase in IFN-γ gene expression at different time points. Previous studies have shown that *in ovo* feeding is helpful for the early immune response. Humphrey and Rudrappa (37) reported that *in ovo* injection of fructose or ribose could promote cell-mediated immunity genes by enhancing the expression of IL-2, IL-12, and IFN-γ. Similar results were also found with the *in ovo* injection of lysine, threonine, methionine, and cystine (38). In addition to carbohydrates and amino acids, El-Senousey et al. (39) have shown that vitamin C could enhance the immune response by significantly decreasing the mRNA level of IL-6, IL-1β, and TNF-α. These findings suggested that *in ovo* feeding could confer a moderate effect of cellular immunity by regulating the production of related cytokines and cellular receptors.

## Conclusions

This study confirmed that the *in ovo* administration of APS does not impact hatchability and may increase the BW at 14 d and 21 d post-hatch. In addition, at most time points, *in ovo* injection of APS at 1 and 2 mg/egg doses could promote the intestinal development and increase the IgA^+^ cells and sIgA content in the intestinal mucosa. Furthermore, *in ovo* injection of APS could alter the expression of several immune-related genes within the ileum. This study reveals that *in ovo* administration of APS could enhance the intestinal development and mucosal immunity in the early stages post-hatch, which provides timely and effective protection for chickens.

At most time points, the villus height (VH) was increased (*P* < 0.05) and the crypt depth (CD) was decreased (*P* < 0.05) in the small intestine of the broilers, with higher VH/CD ratios in the APSL and APSM groups compared with the control group.

## Data Availability Statement

All data generated or analyzed during this study are available from the corresponding author on reasonable request.

## Ethics Statement

The animal study was reviewed and approved by the Jilin Agriculture University Institutional Animal Care and Use Committee (JLAU08201409).

## Author Contributions

Y-nZ and XM designed the study. A-qJ and A-yD assisted with data analysis. S-bY, D-hZ, Y-jQ, W-mL, and PS performed animal tests, interpreted the results, and wrote the manuscript draft. All authors have read and approved the manuscript.

## Conflict of Interest

The authors declare that the research was conducted in the absence of any commercial or financial relationships that could be construed as a potential conflict of interest.

## Publisher's Note

All claims expressed in this article are solely those of the authors and do not necessarily represent those of their affiliated organizations, or those of the publisher, the editors and the reviewers. Any product that may be evaluated in this article, or claim that may be made by its manufacturer, is not guaranteed or endorsed by the publisher.

## Abbreviations

APS: *Astragalus* polysaccharide
BW: body weight
AA+ broilers: Arbor Acres broilers
VH: villus height
CD: depth of crypt
HE staining: hematoxylin-eosin staining
DAB: diaminobenzidine.
